# Impact of Sarcopenia on Acute Kidney Injury after Infrarenal Abdominal Aortic Aneurysm Surgery: A Propensity Matching Analysis

**DOI:** 10.3390/nu13072212

**Published:** 2021-06-27

**Authors:** Ji-Yeon Bang, In-Gu Jun, Jeong-Bok Lee, You-Sun Ko, Kyung-Won Kim, Jun-Hyeop Jeong, Sung-Hoon Kim, Jun-Gol Song

**Affiliations:** 1 Department of Anesthesiology and Pain Medicine, Laboratory for Cardiovascular Dynamics, Asan Medical Center, University of Ulsan College of Medicine, 88 Olympic-ro 43-gil, Songpa-gu, Seoul 05505, Korea; jyounbang@gmail.com (J.-Y.B.); igjun@amc.seoul.kr (I.-G.J.); jhjung000@naver.com (J.-H.J.); shkimans@amc.seoul.kr (S.-H.K.); 2 Department of Clinical Epidemiology and Biostatistics, Asan Medical Center, University of Ulsan College of Medicine, Seoul 05505, Korea; jungboklee@amc.seoul.kr; 3 Department of Radiology, Asan Image Metrics, Asan Medical Center, University of Ulsan College of Medicine, Seoul 05505, Korea; ko.yousun82@gmail.com (Y.-S.K.); kyungwon_kim@amc.seoul.kr (K.-W.K.)

**Keywords:** sarcopenia, abdominal aortic aneurysm, acute kidney injury, postoperative outcome

## Abstract

Background: Sarcopenia contributes to increased morbidity and mortality in patients undergoing surgery for abdominal aortic aneurysms (AAA). However, few reports have demonstrated whether sarcopenia would affect the development of postoperative acute kidney injury (AKI) in these patients. This study aimed to examine whether sarcopenia is associated with AKI and morbidity and mortality after infrarenal AAA operation. Methods: We retrospectively analysed 379 patients who underwent infrarenal AAA surgery. The diagnosis of sarcopenia was performed using the skeletal muscle index, which was calculated from axial computed tomography at the level of L3. The patients were separated into those with sarcopenia (*n* = 104) and those without sarcopenia (*n* = 275). We applied multivariable and Cox regression analyses to evaluate the risk factors for AKI and overall mortality. A propensity score matching (PSM) evaluation was done to assess the postoperative results. Results: The incidence of AKI was greater in sarcopenia than non-sarcopenia group before (34.6% vs. 15.3%; *p* < 0.001) and after the PSM analysis (34.6% vs. 15.4%; *p* = 0.002). Multivariable analysis revealed sarcopenia to be associated with AKI before (*p* = 0.010) and after PSM (*p* = 0.016). Sarcopenia was also associated with overall mortality before (*p* = 0.048) and after PSM (*p* = 0.032). A Kaplan–Meier analysis revealed that overall mortality was elevated patients with sarcopenia before and after PSM than in those without (log-rank test, *p* < 0.001, *p* = 0.022). Conclusions: Sarcopenia was associated with increased postoperative AKI incidence and overall mortality among individuals who underwent infrarenal AAA operation.

## 1. Introduction

Sarcopenia, a syndrome characterised by skeletal muscle loss and dysfunction, is generally observed in elderly patients [1] and has been associated with poor outcomes after major surgeries. Sarcopenia is correlated with elevated mortality after cardiac and noncardiac surgery in previous literatures [2,3,4,5]. The prognostic value of sarcopenia has also been investigated in patients who had undergone surgery for abdominal aortic aneurysms (AAAs), a prevalent disorder in older age [6]. Previous evidence has shown that sarcopenia is independently associated with mortality after AAA treatment [7,8].

Acute kidney injury (AKI) is a complication that is frequent, and adversely affects the prognosis in patients undergoing cardiovascular surgeries [9,10,11]. AKI is known as a significant predicting factor for increased mortality and a lengthened hospital and intensive care unit (ICU) stay in patients undergoing AAA repair [9,12,13,14,15,16]. Although the pathophysiology of AKI is complex and multifactorial, systemic inflammation may play an important role in its development [17]. Similarly, sarcopenia is also associated with an inflammatory condition [1] and inflammation is involved for the mechanism of its development [18]. Thus far, few reports investigated the impact of sarcopenia on AKI among individuals who underwent infrarenal AAA surgery.

This research aimed to investigate the incidence of AKI following infrarenal AAA surgery in patients with and without sarcopenia. We also evaluated the predisposing factors for AKI as well as ICU stay, hospital stay, and overall mortality in patients undergoing infrarenal AAA surgery.

## 2. Methods

### 2.1. Patients

A retrospective analysis was made of 550 consecutive patients who underwent open surgical treatment or endovascular aneurysm repair (EVAR) for AAAs from September 1999 to December 2011. The exclusion criteria were patients aged <18 years (*n* = 1), patients with aneurysms other than infrarenal AAAs (*n* = 77), and patients with ruptured or emergent AAAs (*n* = 93). A total of 379 patients were analysed.

### 2.2. Clinical Data

Data on preoperative, intraoperative, and postoperative outcomes were collected from electronic medical records of patients at our institution. The preoperative variables are as follows: clinical characteristics of patients, such as demographics, body weight, height, body mass index (BMI), and left ventricular ejection fraction; co-morbidities, such as diabetes mellitus, hypertension, coronary arterial disease, cerebrovascular illness, chronic kidney disease (CKD), chronic obstructive pulmonary disease, and atrial fibrillation; preoperative medications, such as beta blockers, aspirin, oral hypoglycaemic agents, and statins; and preoperative laboratory data, such as haemoglobin, estimated glomerular filtration rate (eGFR), serum creatinine (sCr), and serum albumin. Intraoperative variables were as follows: type of surgical procedure, such as open surgery or EVAR; maximal diameter of AAAs; anaesthetic method, such as general, regional, or local anaesthesia; volume and type of fluids, such as total crystalloid infusion, total colloid infusion, or blood products; total urine output; anaesthesia time; aortic clamp time; and lowest mean arterial pressure during operation. Infused colloid agents consisted of albumin, 10% hydroxyethyl starch (HES) 260/0.45 (Pentaspan^TM^), and 6% HES 130/0.4 (Voluven^®^). While AAAs restricted to the aorta were treated with stent grafts, AAAs extending into the iliac arteries were treated with Y-stent grafts. Postoperative data included the hospital stay, ICU stay, and overall mortality.

### 2.3. Definitions

#### 2.3.1. Preoperative Imaging Variables

Sarcopenia was evaluated on abdominal CT with artificial intelligence software (AID-UTM) that was created applying the fully convolutional network (FCN) segmentation method [19]. The expertise operator chose the axial CT slice at the third lumbar vertebra (L3) with the help of coronal recreated views. Selected CT views were routinely segmented to create a margin of full abdominal muscles and measured the abdominal muscle and fat region. An experienced image analyst (Y.K.), who was blind to clinical information, selected axial CT slice at the third lumbar vertebra (L3) inferior endplate level in a semi-automatic manner with the aid of coronal reconstructed images. If there was difficulty identifying the L3 level due to vertebral anatomic variation, an experienced radiologist (K.W.K) determined the exact L3 level.

The skeletal muscle area (SMA), (i.e., psoas, paraspinal, transversus abdominis, rectus abdominis, quadratus lumborum, and internal and external obliques) was identified by applying programmed thresholds (−29 to +150 Hounsfield units) [20]. The visceral and subcutaneous fat regions were defined by employing fat material thresholds (−190 to −30 Hounsfield units) [21]. The SMA was modified by BMI (SMA/BMI), and the result was collectively referred to as the skeletal muscle index (SMI). To obtain reference values for establishing diagnostic cut-off points for sarcopenia, a retrospective evaluation was done in living liver donors aged >18 years who underwent abdominal CT or MRI between January 2008 and December 2011. A total of 1070 living liver donors (755 men and 315 women, age 28.0 ± 8.1 years) were included to evaluate the reference value of the SMI. Acquiescing to the suggestion of the European Working Group on Sarcopenia in Older People (EWGSOP), the cut-off points for sarcopenia were determined at −2 standard deviations (SD) from the mean reference value [22]. As the cut-off for describing sarcopenia, −2 SD from the mean reference value was applied, while the cut-off points of the SMI were 39.6 and 28.6 in men and women, respectively.

#### 2.3.2. Postoperative AKI

Postoperative AKI was classified by the Kidney Disease: Improving Global Outcomes (KDIGO) classification (change of sCr on postoperative days 1–7 from baseline level measured just before the surgery). Stage I was when sCr increased by 0.3 mg/dL or more within 48 h, or by 50–99% above baseline within 7 days; stage II, when sCr increased by 100–199%; and stage III, sCr increased by twofold or 4.0 mg/dL or more beside increase at least 0.5 mg/dL, or renal replacement therapy was necessary.

The main result was the occurrence of AKI based on the KDIGO criteria, which was determined and compared, using a propensity score (PS) matching analysis, between patients with and without sarcopenia who underwent AAA surgery. The secondary outcomes included the effect of sarcopenia on AKI, which was evaluated by elucidating the factors that predisposed the patients to AKI after AAA surgery, and overall mortality, length of hospital stay, and length of ICU stay.

### 2.4. Statistical Analysis

Continuous variables are expressed as means ± SDs or medians and interquartile ranges. For continuous data, analysis was performed using the Student’s t-test or Mann–Whitney Wilcoxon test as appropriate. Categorical parameters are expressed as incidences and percentages and examined applying the chi-square test or Fisher’s exact test. Multiple regression analysis was performed to identify the risk factor of AKI. Parameters with *p* < 0.1 on the univariate were entered in the multivariable analysis. We applied weighted logistic regression assessments to evaluate odds ratios (ORs) and Cox proportional regression assessments for hazard ratios (HRs) of sarcopenia. The Schoenfeld residuals were calculated to test the proportional hazards assumption for each variable. We analysed survival data with the Kaplan–Meier method to evaluate the changes in cumulative survival by the log-rank test. Furthermore, we used propensity score (PS) matching in estimation the effects of sarcopenia on outcome variables, to avoid the selection bias. PS matching were derived at a ratio of 1:1 using greedy algorithms. The variables shown in Table 1 were used to calculate the PS. For model discrimination and calibration, c statistics and Hosmer–Lemeshow statistics (*χ*^2^ = 4.1932, degrees of freedom = 8, *p* = 0.839) were used, respectively. Consecutively, we compared balance in standard parameters between two groups, applying paired *t*-tests for continuous parameters and McNemar’s tests for categorical parameters after matching. In the PS-matched group, the odds ratios of sarcopenia for each result variable in the regression analysis were compared applying generalised estimating equations. A *p*-value of <0.05 was considered statistically significant.

## 3. Results

A total of 379 patients who underwent infrarenal AAA surgery were included. The follow-up period was 3.3 (median) years (1.6–5.2 years, IQR) after AAA operation. Patients were separated into a sarcopenia and a non-sarcopenia group (Table 1). Overall, sarcopenia was observed in 104 patients (27.4%). Of the 379 patients, 212 (55.9%) underwent open repair of AAAs and 167 (44.1%) underwent EVAR (Table 1). Patients with sarcopenia were older (*p* < 0.001) and had higher prevalence of hypertension (*p* = 0.023) and a lower level of preoperative haemoglobin (*p* < 0.001) and albumin (*p* = 0.023) compared with patients without sarcopenia. The prevalence of AKI was 20.6% (*n* = 78) and the overall mortality was 12.9% (*n* = 49). The prevalence of AKI was 15.3% without sarcopenia and 34.6% with sarcopenia patients (*p* < 0.001).

The demographic, preoperative, and intraoperative parameters of PS-matched group (*n* = 104 pairs) were shown in Table 1. After PSM, no statistically significant differences were noted between sarcopenia and non-sarcopenia patients. The incidence of AKI was 15.4% in non-sarcopenia and 34.6% in sarcopenia group after PSM (*p* = 0.002). Multivariable regression assessment revealed that sarcopenia was a risk factor for AKI (OR, 2.19; 95% confidence interval [CI], 1.20–3.98; *p* = 0.010) (Table 2). Preoperative CKD (OR, 3.39; 95% CI, 1.40–8.07; *p* = 0.006) and intraoperative RBC transfusions (OR, 1.17; 95% CI, 1.06–1.30; *p* = 0.002) were also associated with AKI (Table 2).

With regards to the multivariate Cox regression analysis, sarcopenia was related with a higher overall mortality (HR, 1.92; 95% CI, 1.01–3.67; *p* = 0.048), and the association was still seen after PSM (HR, 2.28; 95% CI, 1.08–4.84; *p* = 0.032) (Table 3 and Table 4).

The associations between sarcopenia and the postoperative results are illustrated in Table 4. Sarcopenia was independently associated with AKI before (OR, 2.19; 95% CI, 1.20–3.98; *p* = 0.010) and after PSM (OR, 2.36; 95% CI, 1.19–4.83; *p* = 0.016).

**Figure 1:**
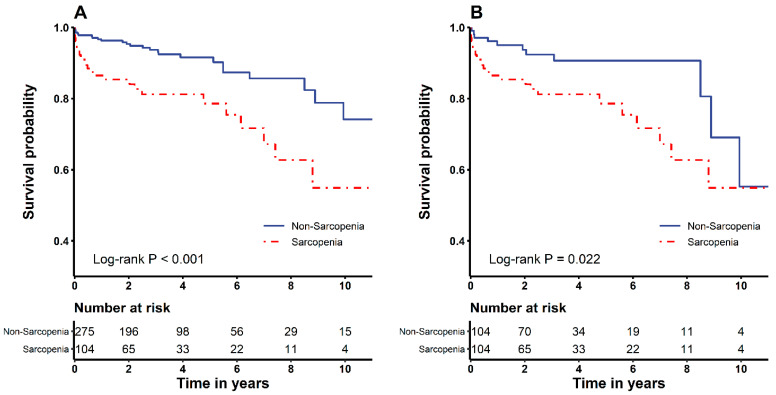
Kaplan–Meier survival curves of the sarcopenia and non-sarcopenia groups before (**A**) and after (**B**) propensity score matching analysis.

The Kaplan–Meier analysis revealed that the overall mortality was significantly higher in sarcopenia patients before (log-rank test, *p* < 0.001) and after PSM assessments (log-rank test, *p* = 0.022) (Figure 1). Hospital stay was considerably extended in the sarcopenia group compared to the non-sarcopenia group before (8 [6,7,8,9,10] vs. 9 [6.5–15] days; *p* = 0.001) and after PSM (8 [5.5–9] vs. 9 [6.5–15] days; *p* = 0.003). ICU stay was also considerably longer in the sarcopenia group compared to the non-sarcopenia group before (2 [2,3] vs. 2 [2–3.5] days; *p* = 0.017) and after PSM (2 [2,3] vs. 2 [2–3.5] days, *p* = 0.026) (Table 4).

## 4. Discussion

This study demonstrated that sarcopenia was associated with AKI in patients who had undergone infrarenal AAA surgery. The association remained even after PSM analysis of 104 pairs of patients. Furthermore, sarcopenia was significantly associated with a higher risk of overall mortality in patients who underwent infrarenal AAA surgery, and hospital stay and ICU stay were significantly longer in sarcopenia patients than in patients without sarcopenia, before and after PSM analysis.

Sarcopenia, characterised by loss of skeletal muscle mass and deterioration in muscle work and physical performing, has recently gained attention because of its association with adverse outcomes. Although sarcopenia has been known to be a marker of frailty, sarcopenia is related to short-term or overall mortality after AAA surgery [23]. For both endovascular and open repair of AAA, sarcopenia was related to increased mortality [7,8]. The meta-analysis demonstrated that low skeletal muscle mass was associated with increased mortality in open or EVAR for AAA patients (HR 1.66, 95% CI 1.15–2.40; *p* = 0.007) [24,25,26,27]. After subgroup analysis, patients with EVAR without sarcopenia revealed minimal survival improvement (HR 1.86, 95% CI 1.00–3.43; *p* = 0.05) [8,28]. Studies in patients with EVAR found no significant difference in peri-operative mortality (RD 0.04, 95% CI −0.13 to 0.21) and morbidity (OR 1.58, 95% CI 0.90–2.76; *p* = 0.11) between patients with and without low skeletal muscle mass [29,30]. Therefore, sarcopenia can be used to stratify the risk of AAA patients. However, it is difficult to use the reference values, because the diagnostic criteria for sarcopenia are different.

In our study, sarcopenia was defined using the cut-off points of the SMI, which were determined at −2 SD from the mean reference value based on data from a healthy population, and these cut-off points in men and women were 39.6 and 28.6, respectively. The incidence of sarcopenia was 26.2% in men, 37.5% in women, and 27.4% in all patients. If our study used the definition of sarcopenia based on the criteria determined by Prado et al., which are widely used, the incidence of sarcopenia would have increased to 81.8% [31]. Therefore, the criteria for sarcopenia based on the cut-off value of the SMI in our healthy population would be more appropriate for use in clinical research, as they better represent the general Asian population.

According to a previous study, sarcopenia is linked with decreasing kidney function, which sequentially decreases the eGFR [32]. Another study demonstrated that sarcopenia is associated with kidney damage [33]. The exact reason for the prevalence of AKI in patients with sarcopenia has not been determined. However, previous studies have demonstrated that inflammatory pathway activation following chronic inflammation is an important contributor to sarcopenia [34,35]. In the same manner, inflammation is also associated with the development of AKI. In this study, sarcopenia might have influenced the incidence of AKI in patients who underwent infrarenal AAA surgery.

The primary goal of our study was to verify the occurrence of AKI in patients with sarcopenia who underwent infrarenal AAA surgery; this was found to be 34.6%. The patients with sarcopenia were older and more likely to have a higher prevalence of hypertension and a lower level of preoperative albumin. After reducing these perioperative confounding factors by PSM analysis, the higher prevalence of AKI in the sarcopenia compared with non-sarcopenia group remained statistically significant.

Sarcopenia has been reported in CKD patients on haemodialysis [36]. A recent study has demonstrated that sarcopenia was common in patients with advanced stages of CKD as well as in patients with an early stage of CKD who were not yet on haemodialysis [36]. Consistent with this study, our results demonstrated that preoperative CKD was also a risk factor for the development of postoperative AKI. One possible mechanism that causes sarcopenia in CKD patients is that CKD is linked with reduced regeneration [37]. Factors such as accretion of oxidative stress, chronic inflammation, malnutrition, and uremic toxins are also associated with increased catabolism in CKD patients [38,39,40,41]. Taken together, these findings indicate that sarcopenia is associated with CKD, which appears to be associated with postoperative AKI.

In this study, sarcopenia was associated with postoperative adverse outcomes, such as a higher risk of overall mortality and prolonged hospital stay and ICU stay. Consistent with our results, Newton et al. also demonstrated that sarcopenia was linked with worse survival among patients who underwent EVAR [7]. Furthermore, Lindstrom et al. showed that the psoas muscle area and quality, evaluated by the L2-L3 psoas muscle region and density, are reliable, feasible, and impartial prognosticators of mortality in AAA patients [7].

There are several limitations in our study. First, since the study was retrospective, causality could not be determined. Although we applied statistical methodologies and a PSM assessment, the impacts of remaining confounding issues might still be present and could not be completely abolished. Second, since our research was conducted in a single centre, our perioperative manage practices might have altered the occurrence of AKI. Therefore, generalisation of our results might not be possible. Third, applying the sCr level as the major principle in identifying AKI may have led us to miss certain patients who were in danger of AKI. Lastly, as we only registered patients who underwent AAA surgery, these findings cannot be generalised and must be interpreted with caution.

In conclusion, this study revealed that sarcopenia was associated with an increase in postoperative AKI incidence, overall mortality, and hospital and ICU stay in patients who underwent infrarenal AAA surgery. The evaluation of sarcopenia may be valuable to discriminate poor outcomes after infrarenal AAA surgery. 

## Figures and Tables

**Table 1:** Patient demographics and preoperative and intraoperative characteristics according to sarcopenia.

	Before Propensity Score Matching				After Propensity Score Matching		
	Total (*n* = 379)	No Sarcopenia (*n* = 275)	Sarcopenia (*n* = 104)	*p* Value	No sarcopenia (*n* = 104)	Sarcopenia (*n* = 104)	*p* Value
**Patients’ demographics**							
Age (yr)	69.0 (64.0–74.0)	67.0 (63.0–73.0)	74.0 (69.0–78.0)	<0.001	74 (68–76)	74 (69–78)	0.293
Sex, male	339 (89.4)	250 (90.9)	89 (85.6)	0.187	91 (87.5)	89 (85.6)	0.839
Body mass index (kg/m^2^)	23.8 ± 3.4	24.7 ± 3.1	21.5 ± 3.1	<0.001	-	-	-
Diabetes	62 (16.4)	46 (16.7)	16 (15.4)	0.873	15 (14.4)	16 (15.4)	1.000
Hypertension	272 (71.8)	188 (68.4)	84 (80.8)	0.023	83 (79.8)	84 (80.8)	1.000
Coronary arterial disease	118 (31.1)	87 (31.6)	31 (29.8)	0.827	34 (32.7)	31 (29.8)	0.765
CVD	40 (10.6)	27 (9.8)	13 (12.5)	0.568			-
COPD	107 (28.2)	71 (25.8)	36 (34.6)	0.116	-	-	-
CKD	31 (8.2)	20 (7.3)	11 (10.6)	0.402	8 (7.7)	11 (10.6)	0.630
Beta blocker	119 (31.4)	83 (30.2)	36 (34.6)	0.480	-	-	-
Statin	134 (35.4)	102 (37.1)	32 (30.8)	0.304	-	-	-
Haemoglobin (g/dL)	13.3 (12.1–14.2)	13.5 (12.4–14.4)	12.4 (10.9–13.8)	<0.001	12.6 (11.4–13.8)	12.4 (10.9–13.8)	0.481
Albumin (g/dL)	3.8 (3.4–4.0)	3.8 (3.5–4.0)	3.6 (3.2–3.9)	0.002	3.7 (3.4–4.0)	3.6 (3.2–3.9)	0.326
Creatinine (mg/dL)	0.8 ± 0.2	0.8 ± 0.2	0.8 ± 0.2	0.119	-	-	-
eGFR (ml/min/1.73 m^2^)	69.0 (60.0–85.9)	71.0 (60.0–86.0)	66.0 (60.0–80.8)	0.126	70.0 (60–82.2)	66.0 (60.0–80.8)	0.605
**Intraoperative variables**							
**Treatment**				0.535			1.0
Open surgery	212 (55.9)	157 (57.1)	55 (52.9)		54 (51.9)	55 (52.9)-	
EVAR	167 (44.1)	118 (42.9)	49 (47.1)		50 (48.1)	49 (47.1)	
Maximal diameter	5.7 (5.0–6.5)	5.7 (5.0–6.5)	5.8 (5.0–6.5)	0.212	6.0 (5.0–6.8)	5.8 (5.0–6.5)	0.975
**Anaesthesia type**				0.574			0.379
General	314 (82.8)	230 (83.6)	84 (80.8)		84 (80.8)	84 (80.8)	
Regional	36 (9.5)	27 (9.8)	9 (8.7)		14 (13.5)	9 (8.7)	
Local	29 (7.7)	18 (6.5)	11 (10.6)		6 (5.8)	11 (10.6)	
Crystalloid (mL)	3100 (1700–4300)	3050 (1700–4300)	3100 (1900–4200)	0.982	-	-	-
Colloid (mL)	600 (100–1000)	600 (125–1000)	625 (100–1000)	0.853	600 (150–1000)	625 (100–1000)	0.767
Red blood cell transfusion (Units)	2 (0–4)	2 (0–4)	2 (0–4)	0.090	2 (0–4)	2 (0–4)	0.828
Fresh frozen plasma transfusion (Units)	0 (0–0)	0 (0–0)	0 (0–0)	0.391	-	-	-
Total urine output (mL)	680 (400–1070)	700 (430–1082)	635 (340–1030)	0.179	-	-	-
Anaesthesia time (min)	273 (209.5–340)	273 (208–336.5)	276.5 (219.5–357)	0.503	261.5 (207–352)	276.5 (219.5–357)	0.706
Aortic clamp time (min)	55 (0–94.5)	55 (0–93.5)	48 (0–95)	0.631	41.5 (0–95)	48.0 (0–95)	0.989
Lowest mean arterial pressure (mmHg)	65.3 (61–72)	65.7 (61.3–71.8)	65.0 (59.7–73)	0.513	67 (62–72)	65 (60–73)	0.327

Values are expressed as the mean ± standard deviation or median (1st quartile and 3rd quartile) for continuous variables and *n* (%) for categorical variables. CVD, cerebrovascular disease; COPD, chronic obstructive pulmonary disease, CKD, Chronic kidney disease; eGFR, estimated glomerular filtration rate; EVAR, Endovascular aneurysm repair. EVAR, Endovascular aneurysm repair.

**Table 2:** Multivariable analysis of risk factors associated with acute kidney injury.

	Univariate			Multivariable		
	OR	95% CI	*p* Value	OR	95% CI	*p* Value
Sarcopenia	2.94	1.74–4.95	<0.001	2.19	1.20–3.98	0.010
Age	1.06	1.02–1.10	0.003	1.03	0.99–1.07	0.134
Sex, male	0.80	031–1.79	0.611			
Diabetes	1.03	0.51–1.96	0.934			
Preoperative CKD	4.92	2.30–10.58	<0.001	3.39	1.40–8.07	0.006
Maximal diameter	1.19	1.00–1.41	0.051			
EVAR	0.70	0.42–1.16	0.171			
Preoperative haemoglobin	0.72	0.62–0.83	<0.001			
Preoperative albumin	0.36	0.21–0.59	<0.001	0.58	0.33–1.02	0.057
Infused colloid	1.00	1.00–1.00	0.165			
Diuretics	1.18	0.92–1.50	0.178			
Intraoperative RBC transfusion	1.21	1.11–1.34	<0.001	1.17	1.06–1.30	0.002
Aortic clamping time	1.00	1.00–1.00	0.725			
Lowest intraoperative MBP	1.00	1.00–1.01	0.919			
Anaesthetic time	1.00	1.00–1.01	0.221			

CKD, Chronic kidney disease; EVAR, Endovascular aneurysm repair; RBC, Red blood cell.

**Table 3:** Cox proportional hazard analysis of risk factors associated with overall mortality.

	Univariate			Multivariable		
	HR	95% CI	*p* Value	HR	95% CI	*p* Value
Sarcopenia	2.72	1.55–4.77	0.005	1.92	1.01–3.67	0.048
Age	1.11	1.06–1.16	<0.001	1.09	1.04–1.14	0.008
Sex, male	1.67	0.78–3.56	0.187			
DM	1.81	0.92–3.56	0.865	2.25	1.12–4.51	0.022
Hypertension	1.379	0.72–2.65	0.334			
Preoperative CKD	3.52	1.75–7.09	0.004	2.72	1.13–6.57	0.026
Maximal diameter	1.08	0.89–1.30	0.437			
EVAR	1.66	0.92–3.02	0.947	1.60	0.86–2.97	0.136
Preoperative haemoglobin	0.81	0.69–0.94	0.007	1.18	0.96–1.45	0.119
Preoperative albumin	0.32	0.20–0.51	<0.001	0.31	0.18–0.54	<0.001
Diuretics	0.93	0.70–1.23	0.608			
Intraoperative RBC transfusion	1.08	0.99–1.17	0.084			
Aortic clamping time	1.00	0.99–1.00	0.500			
Lowest intraoperative MBP	1.01	0.98–1.04	0.603			
Anaesthetic time	1.00	1.00–1.00	0.670			

MELD, Model for End-stage Liver Disease.

**Table 4:** Clinical outcomes adjusted by sarcopenia.

		**Multivariate**			**PS Matching**		
		**Event/N (%)**	**OR (95% CI)**	***p* Value**	**Event/N (%)**	**OR (95% CI)**	***p* Value**
AKI	No sarcopenia	42/275 (15.3)	1		16/104 (15.4)	1	
	Sarcopenia	36/104 (34.6)	2.19 (1.20–3.98)	0.010	36/104 (34.6)	2.36 (1.19 –4.83)	0.016
		**Event/N (%)**	**HR (95% CI)**	***p* Value**	**Event/N (%)**	**HR (95% CI)**	***p* Value**
Overall mortality	No sarcopenia	25/275 (9.1)	1		11/104 (10.6)	1	
	Sarcopenia	24/104 (23.1)	1.92 (1.01–3.67)	0.048	24/104 (23.1)	2.28 (1.08–4.84)	0.032
		**Before PS Matching**			**After PS Matching**		
		**Median (IQR)**		***p* Value**	**Median (IQR)**		***p* Value**
Hospital stay	No sarcopenia	8 (6–10)		0.001	8 (5.5–9)		0.003
	Sarcopenia	9 (6.5–15)			9 (6.5–15)		
ICU stay	No sarcopenia	2 (2–3)		0.017	2 (2–3)		0.026
	Sarcopenia	2 (2–3.5)			2 (2–3.5)		

PS, propensity score; OR, odds ratio; AKI, acute kidney injury; HR, hazard ratio; IQR, interquartile range; ICU, intensive care unit.

## Data Availability

The data presented in this study are available on request from the corresponding author. The data are not publicly available due to conditions of the ethics committee of our university.
